# Association Between Mental Health Status and Nicotine Dependence Among Adult Cigarette Smokers Attending Primary Health Care Centers in Taif City, Saudi Arabia

**DOI:** 10.7759/cureus.89532

**Published:** 2025-08-07

**Authors:** Ahmed Alnashri, Asheqah Albalwi, Nesrin K Abd El-Fatah

**Affiliations:** 1 Preventive Medicine, Postgraduate Training Program for Preventive Medicine, Taif, SAU; 2 Public Health Administration, Ministry of Health, Taif, SAU; 3 Public Health, High Institute of Public Health, Alexandria University, Alexandria, EGY

**Keywords:** cigarette smoking, depression, mental health, nicotine dependence, primary healthcare centers, saudi arabia, smoking cessation

## Abstract

Background: Smoking cigarettes is increasingly becoming a strong focus of concern for public health in Saudi Arabia, especially with the rise in mental health disorders. This research explores how depression, anxiety, and stress are related to the level of dependence on nicotine.

Methods: This cross-sectional study involved 310 adult smokers and utilized face-to-face interviews to collect data on participants' sociodemographic and lifestyle factors, nicotine dependence through the Fagerstrom Test of Nicotine Dependence (FTND), and mental health status using a validated Arabic version of the short form of the Depression Anxiety Stress Scales (DASS-21).

Results: The study sample consisted mostly of males (n=232, 74.8%), with a mean age of 42.3 years. Nicotine dependence was significantly elevated, with high dependence noted in 52.3% (n=162) of participants. Mental health symptoms were notable and severe among respondents: 44.8% (n=139) reported severe depression, 29.7% (n=92) had severe anxiety, and 38.1% (n=118) experienced stress of considerable severity. Multivariate analysis suggested strong correlations between nicotine dependence and mental health outcomes, noting that high dependence increased the odds of depression by 30.36 times (95% CI: 7.65-120.46) and stress by 27.20 times (95% CI: 6.20-119.39). Having a chronic disease (61.9% of participants, n=192) was significantly associated with poorer mental health outcomes (depression OR = 13.81, 95% CI: 1.24-154.10). Furthermore, participants with income less than 3,000 SAR/month exhibited considerably higher anxiety than their higher income counterparts (OR = 0.16, 95% CI: 0.04-0.75 for the 3,000-6,000 SAR group).

Conclusion: This study highlights the relationship between nicotine dependence and mental disorders in Saudi smokers. Such mental disorders were further linked to chronic illnesses and socioeconomic elements such as income. Moreover, there is an apparent need for the Saudi primary healthcare system to incorporate comprehensive mental health services alongside smoking cessation services at the primary care level, advancing healthcare in Saudi Arabia.

## Introduction

Cigarette smoking remains a major public health burden worldwide, negatively impacting health and increasing the risk of non-communicable diseases, such as heart diseases, lung conditions, and cancer [1,2]. Smoking also has significant effects on mental health. Research indicates that individuals with mental health disorders, particularly depression, anxiety, or stress, tend to smoke more and have increased difficulty quitting [3,4]. This creates a detrimental cycle in which mental health challenges lead to increased smoking, and smoking worsens mental health issues [5]. In Saudi Arabia, there is a high smoking prevalence among both adults and youth [6]. Social and cultural factors, including stress, peer pressure, and the belief that cigarettes aid relaxation, encourage these habits among Saudi smokers [7]. Primary healthcare centers (PHCs) play an essential role in the Saudi healthcare system [8], assisting patients willing to quit smoking and those with mental health problems. However, there seems to be a gap in the literature regarding the relationship between mental health and nicotine dependence, particularly in Saudi cities like Taif. While bupropion is commonly included in international guidelines as a first-line recommendation for smoking cessation treatment [9,10], it continues to be underutilized within Saudi Arabia, specifically in primary healthcare settings. It is practically offered to no one, despite evidence indicating its particular suitability for individuals trying to quit smoking who also have comorbid depression. In such situations, bupropion can provide valuable treatment by lessening withdrawal and craving symptoms while simultaneously improving mood [11,12]. This absence of treatment use illustrates the lack of attention to smokers with mental illness and the symptoms they commonly face, and highlights the need to focus on effective treatment, such as bupropion, and the reasons for its underutilization in Saudi PHCs.

Although several international studies have examined the connection between mental health and smoking, there is a significant gap in local data from Saudi Arabia, and more specifically, from Taif City, which appears to present high risks such as stress, mental health issues, and smoking, as well as peer pressure among adolescents [13]. Prior studies indicate that factors like financial strain, burnout, unemployment, and low educational attainment are socioeconomic drivers of poor mental health and smoking behavior [12]. Hence, this study aims to explore the mental health status in relation to the level of nicotine dependence among adult smokers attending PHCs in Taif City.

This study aims to investigate the association between mental health status and nicotine dependence among adult cigarette smokers attending PHCs in Taif City, Saudi Arabia. Specifically, we hypothesize that higher levels of nicotine dependence are significantly associated with worse mental health outcomes (depression, anxiety, and stress). Additionally, we explore the roles of socioeconomic factors, chronic diseases, and lifestyle behaviors in moderating this relationship. The findings would help in the formulation of public health policies and strengthen the services intended for mental healthcare by promoting proper utilization of bupropion among Saudi citizens at high risk.

## Materials and methods

Study design and settings

This cross-sectional study was carried out between January 1 and 31, 2025, on 4 out of 19 PHCs in Taif City, which were randomly selected to ensure geographical and demographic representativeness.

Study population

The study population included male and female adult cigarette smokers (defined as individuals aged 18 or older) visiting these centers during the study period. Participants had to be registered at the selected PHCs and report having smoked cigarettes in the past 30 days to be included. Exclusion criteria were defined as non-Saudi citizens, smokers of other types of tobacco products (such as shisha and e-cigarettes), and individuals under the age of 18. The sample size was calculated using Epi Info 6 software, considering the prevalence of depression among smokers to be 78.7% [14], 95% confidence level, and 5% margin of error. The sample was estimated to be 258 participants. Considering a possible non-response rate of 20%, the sample was increased to 310 participants. The predetermined sample was proportionally allocated among the PHCs. Participants were randomly recruited during their PHC visits each day. The response rate was approximately 94%.

Data collection methods

The data were collected using structured, face-to-face, questionnaire-based interviews conducted by trained data collectors. The questionnaire was subdivided into an inclusivity screening section, demographic data, smoking records, nicotine dependence, chronic diseases, lifestyle and mental health factors, and socioeconomic data.

Variable measurements

Mental health was assessed using the validated Arabic version of the Depression, Anxiety, and Stress Scale-21 (DASS-21) [15]. It consists of 21 items (7 per subscale) rated on a 4-point Likert scale (0 to 3). Severity levels were categorized based on standardized cutoff scores [16] as follows: Depression: Normal (0-9), Mild (10-13), Moderate (14-20), Severe (21-27), and Extremely Severe (≥28); Anxiety: Normal (0-7), Mild (8-9), Moderate (10-14), Severe (15-19), and Extremely Severe (≥20); Stress: Normal (0-14), Mild (15-18), Moderate (19-25), Severe (26-33), and Extremely Severe (≥34). The reliability of the Arabic version of the DASS-21 was assessed with Cronbach's α = 0.83.

Nicotine dependence was assessed using the Arabic version of the Fagerstrom Test for Nicotine Dependence (FTND) [17]. It consists of 6 questions (3 multiple-choice and 3 yes/no questions), and the total score ranged from 0 to 10, indicating low to high dependence [18] as follows: Low dependence: 0-2, Low to moderate dependence: 3-4, Moderate dependence: 5-6, and High dependence: 7-10. This tool demonstrated great internal consistency (α = 0.874) according to Cronbach’s alpha [17,18].

Lifestyle factors included smoking history (age at initiation, attempts to quit, duration of smoking, and years of smoking), physical activity, and Body Mass Index (BMI). The level of physical activity was measured using the International Physical Activity Questionnaire short form (IPAQ-SF) [19], which has been validated in several countries. The IPAQ-SF results were classified as low, moderate, or high physical activity levels [20], as follows: Sedentary: No physical activity for at least 10 minutes per week; Insufficiently Active: Some activity, but not enough to be considered active; Active: Meeting any of the following-vigorous activity ≥3 days/week for ≥20 minutes, moderate or walking activity ≥5 days/week for ≥30 minutes, or total activity ≥150 minutes/week; Very Active: Vigorous activity ≥5 days/week for ≥30 minutes, or vigorous activity ≥3 days/week combined with moderate or walking activity ≥5 days/week.

Weight and height were assessed using standardized protocols. Weight was measured with a calibrated digital scale, and height was measured with a stadiometer. BMI was calculated as weight in kilograms divided by height in meters squared (kg/m²) and classified according to the World Health Organization criteria: underweight (<18.5 kg/m²), normal weight (18.5-24.9 kg/m²), overweight (25-29.9 kg/m²), and obesity (≥30 kg/m²). Other variables included demographics such as age, gender, marital status, living situation, employment, education, and monthly income. The self-reported history of chronic diseases and medication usage was also assessed.

Statistical analysis

All statistical analyses were carried out using IBM SPSS Statistics for Windows, Version 27 (Released 2020; IBM Corp., Armonk, New York, United States). Descriptive statistics included computing frequencies and percentages for categorical variables such as gender, marital status, education level, presence of chronic disease, level of nicotine dependence, severity of mental health issues, level of physical activity, and BMI category. Means and standard deviations were calculated for other variables, including age, years of smoking, DASS-21 subscale scores, FTND total score, and BMI. Univariate analyses explored the relationships between demographic variables, smoking history, level of nicotine dependence, chronic disease, lifestyle factors, and scores on the DASS subscales.

Non-parametric methods were used for analysis. Mann-Whitney U tests were applied to independent variables containing two categories, whereas groups with more than two categories were assessed using Kruskal-Wallis H tests to compare median DASS-21 scores among the different groups. To determine mental health outcomes assessed as binary (Normal vs. any symptoms of depression, anxiety, and stress), multivariate binary logistic regression models were conducted for each mental health subscale. The Omnibus Tests of Model Coefficients were used to evaluate the significance of the overall logistic regression models. Model fit was determined by -2 Log Likelihood, and predictive power was assessed using Cox and Snell R Square, Nagelkerke R Square, and the classification table showing sensitivity and specificity, demonstrating the model’s accuracy. Statistical significance was defined as p < 0.05 for all analyses. All demographic, smoking-related, health, and lifestyle variables were first assessed in univariate analyses. Then, multivariate logistic regression models were constructed incorporating variables that exhibited statistical significance in the preceding univariate comparisons.

Ethical considerations

The research received institutional ethical approval from the Research Ethics Committee of Taif Health Affairs, Ministry of Health, Saudi Arabia (IRB: H-02-T-123, Number 2025-E-08). Written informed consent was obtained from all participants after explaining the study objectives, procedures, and their right to withdraw at any time without consequences. Confidentiality was strictly maintained, and all data were anonymized to protect participant privacy.

## Results

Table 1 summarizes the demographic profile of the 310 adult cigarette smokers in the study. It highlights characteristics such as age distribution (40.0% aged 50-69 years), gender composition (74.8% male), and marital status (62.6% married). It also shows that most participants reported having at least one chronic disease (61.9%) and were either overweight (56.5%) or obese (27.7%). Furthermore, the table presents the prevalence of having “Any Symptoms” of mental health disorders, revealing high rates of symptoms across all three domains: 95.8% for depression, 83.9% for anxiety, and 89.4% for stress.

**Table 1:** Demographic Characteristics of Adult Cigarette Smokers Attending PHCCs in Taif City, Saudi Arabia (N = 310) PHCC: primary health care centers.

Variable	Category	Frequency (n)	Percent (%)
Age classification	18-29 years	69	22.3
	30-49 years	117	37.7
	50-69 years	124	40.0
Gender	Female	78	25.2
	Male	232	74.8
Marital status	Divorced	44	14.2
	Married	194	62.6
	Single	41	13.2
	Widowed	31	10
Living status	Alone	59	19
	With family	251	81
Employment status	Employed full-time	113	36.5
	Employed part-time	80	25.8
	Retired	89	28.7
	Unemployed	28	9
Education level	Postgraduate degree	93	30
	Primary school	6	1.9
	Secondary school	79	25.5
	University degree	132	42.6
Monthly income	< 3,000 SAR	65	21
	3,000–6,000 SAR	113	36.5
	6,001–10,000 SAR	108	34.8
	> 10,000 SAR	24	7.7
Chronic disease	Yes	192	61.9
Physical activity level	Active	144	46.5
	Insufficiently active	142	45.8
	Sedentary	24	7.7
BMI category	Normal weight	49	15.8
	Overweight	175	56.5
	Obese	86	27.7
Depression	Yes	297	95.8
Anxiety	Yes	260	83.9
Stress	Yes	277	89.4

Table 2 summarizes the smoking patterns and nicotine dependence levels among participants. It shows that a large proportion began smoking during their teenage years (34.8%) and that the average smoking duration was over 26 years. Over half (57.1%) had attempted to quit before. Still, a significant majority exhibited moderate (28.7%) to high (52.3%) nicotine dependence based on the FTND, characterized by smoking soon after waking (73.2% within 30 minutes) and high daily consumption (72.9% smoking >20 cigarettes/day).

**Table 2:** Smoking History and Nicotine Dependence Characteristics of Adult Cigarette Smokers Attending PHCCs in Taif City, Saudi Arabia (N = 310) N = Total number of participants. Years Smoking: Mean ± SD = 26.24 ± 14.12 years, Range = 1-54 years. "Most Difficult Cigarette" refers to which cigarette a smoker would hate most to give up, typically indicative of the strongest craving as per the Fagerström Test for Nicotine Dependence. PHCC: primary health care centers.

Variable	Category	Frequency (n)	Percent (%)
Start smoking age	Child (<13)	51	16.5
	Teen (13-17)	108	34.8
	Young adult (18-20)	73	23.5
	Adult (21-24)	78	25.2
Attempted to quit	Yes	177	57.1
Number of quit attempts	1–2 times	65	21
	3–5 times	59	19
	More than 5 times	53	17.1
	None	133	42.9
Longest quit duration	Less than 1 month	59	19
	1–6 months	61	19.7
	More than 6 months	57	18.4
	Never quit	133	42.9
First cigarette after waking	Within 5 minutes	93	30
	6–30 minutes	134	43.2
	31–60 minutes	62	20
	After 60 minutes	21	6.8
Smoking in restricted areas	Yes	175	56.5
Most difficult cigarette	The first one in the morning	208	67.1
	Any other	102	32.9
Cigarettes per day	More than 30	115	37.1
	21–30	111	35.8
	11–20	62	20
	10 or fewer	22	7.1
Smoking more in the morning	Yes	188	60.6
Smoking despite illness	Yes	152	49
Nicotine dependence level	High	162	52.3
	Moderate	89	28.7
	Low to moderate	46	14.8
	Low	13	4.2

Table 3 presents the comparison of DASS-21 subscale scores for depression, anxiety, and stress across various demographic, smoking, health, and lifestyle factors. Statistically significant differences (p ≤ 0.05) in mental health scores were observed across multiple variables, including age, marital status, living status, employment, education, monthly income, chronic disease status, nicotine dependence level, physical activity level, and BMI category. Notably, higher levels of nicotine dependence and the presence of chronic diseases were associated with higher median scores across all three mental health subscales. Factors such as age and socioeconomic status also showed significant associations, although smoking age and attempts to quit did not consistently show significant differences across all mental health outcomes.

**Table 3:** Comparison of DASS-21 Subscale Scores Among Adult Cigarette Smokers Values represent Median (IQR). Mann-Whitney U test was used for dichotomous variables. The Kruskal-Wallis H test was used for variables with more than two categories. * Significant at p ≤ 0.05.

Variable	Category	Depression Score Median (Q1-Q3)	Depression Test	Depression P-value	Anxiety Score Median (Q1-Q3)	Anxiety Test	Anxiety P-value	Stress Score Median (Q1-Q3)	Stress Test	Stress P-value
Age classification	18-29 years	14.00 (11.0-17.5)	H = 113.8	<0.001*	7.0 (3.0-11.0)	H = 96.1	<0.001*	16.50 (12.0-19.5)	H = 106.3	<0.001*
	30-49 years	20 (17.0-23.0)			14.00 (11.0-19.0)			24.00 (19.0-27.0)		
	50-69 years	22.50 (21.0-24.0)			17.00 (14.0-20.0)			26.00 (23.0-29.0)		
Gender	Female	21.00 (18.0-24.0)	U = 7733.50	0.054	15.00 (10.0-19.00)	U = 8358.50	0.313	25.00 (21.0-28.0)	U = 7822.50	0.073
	Male	20.00 (17.0-23.0)			14.00 (10.0-19.00)			23.00 (18.0-27.0)		
Marital status	Divorced	20.00 (17.0-24.0)	H = 45.95	<0.001*	15.50 (12.0-19.0)	H = 37.19	<0.001*	25.50 (21.2-28.0)	H = 44.92	<0.001*
	Married	21.00 (18.0-24.0)			15.00 (11.0-19.0)			25.00 (19.0-27.2)		
	Single	14.00 (11.5-18.0)			9.00 (3.50-12.0)			17.00 (12.0-21.0)		
	Widowed	21.00 (20.0-23.0)			16.00 (13.0-19.0)			25.00 (21.0-28.0)		
Living status	Alone	21.00 (19.0-24.0)	U = 5641.00	0.004*	17.00 (12.0-20.0)	U = 5850.00	0.012*	25.00 (22.0-28.0)	U = 6170.00	0.046*
	With family	20.00 (17.0-23.0)			14.00 (9.0-18.0)			23.00 (18.0-27.0)		
Employment status	Employed full-time	20.00 (17.00-23.00)	H = 55.72	<0.001*	14.00 (10.00-19.00)	H = 42.10	<0.001*	24.00 (18.50-27.00)	H = 47.71	<0.001*
	Employed part-time	19.00 (15.25-22.00)			13.00 (7.25-17.00)			22.00 (17.00-27.00)		
	Retired	23.00 (20.00-24.00)			17.00 (14.00-20.00)			26.00 (23.00-29.00)		
	Unemployed	14.50 (11.00-17.75)			9.50 (3.25-13.75)			17.50 (12.00-21.75)		
Education level	Postgraduate degree	20.00 (18.00-23.00)	H = 16.07	0.001*	16.00 (13.00-19.00)	H = 9.75	0.021*	25.00 (19.00-28.00)	H = 10.94	0.012*
	Primary school	13.50 (9.00-16.50)			11.50 (5.25-12.50)			17.00 (11.50-19.50)		
	Secondary school	22.00 (19.00-24.00)			15.00 (11.00-19.00)			25.00 (20.00-28.00)		
	University degree	20.00 (16.00-23.00)			13.00 (9.00-19.00)			23.00 (17.25-27.00)		
Monthly income (SAR)	< 3.000 SAR	17.00 (13.0-21.0)	H = 26.68	<0.001*	12.00 (6.50-16.5)	H = 16.77	0.001*	19.00 (16.0-25.0)	H = 21.07	<0.001*
	3.000–6,000	21.00 (18.0-24.0)			15.00 (12.0-19.0)			25.00 (20.0-28.0)		
	6.001–10,000	21.00 (19.0-24.0)			16.00 (12.0-19.0)			25.00 (21.0-28.0)		
	>10.000 SAR	19.00 (17.0-21.0)			14.00 (9.0-19.0)			23.50 (19.0-27.0)		
Start smoking age classification	Child (<13)	20.00 (18.0-23.0)	H = 0.27	0.966	15.00 (10.0-19.0)	H = 0.64	0.887	24.00 (19.0-26.0)	H = 2.29	0.515
	Teen (13-17)	20.50 (17.0-23.0)			14.00 (10.0-19.0)			23.00 (19.0-26.0)		
	Young adult (18-20)	20.00 (17.0-23.0)			15.00 (12.00-19.00)			25.00 (19.0-29.0)		
	Adult (21-24)	20.00 (17.0-24.0)			14.00 (8.0-19.0)			25.00 (18.7-28.0)		
Attempted to quit before	Yes	20.00 (17.0-23.0)	U = 9970.50	0.021*	14.00 (10.0-18.5)	U = 10562.50	0.121	24.00 (18.0-27.0)	U = 11092.00	0.384
	No	21.00 (18.0-24.0)			15.00 (10.5-19.0)			24.00 (19.5-28.0)		
Chronic diseases	Yes	21.00 (17.0-24.0)	U = 8369.50	<0.001*	15.00 (11.0-19.0)	U = 9425.50	0.013*	25.00 (20.0-28.0)	U = 9046.00	0.003*
	No	19.00 (15.7-22.0)			13.00 (8.7-19.0)			22.00 (16.7-26.0)		
Nicotine dependence level	Low	7.00 (4.00-11.0)	H = 201.83	<0.001*	0.00 (0.00-2.00)	H = 181.74	<0.001*	5.00 (0.00-12.0)	H = 168.77	<0.001*
	Low to moderate	12.50 (10.00-15.0)			6.50 (4.00-10.0)			15.00 (13.0-18.0)		
	Moderate	19.00 (17.0-20.0)			13.00 (10.0-15.0)			22.00 (18.00-25.0)		
	High	23.00 (21.0-25.0)			18.00 (15.0-21.0)			26.00 (24.0-30.0)		
Physical activity level	Active	19.00 (15.0-22.0)	H = 23.81	<0.001*	13.00 (8.00-17.0)	H = 23.47	<0.001*	21.00 (16.0-26.0)	H = 27.28	<0.001*
	Insufficiently active	21.00 (19.0-24.0)			16.00 (12.0-19.25)			25.00 (21.0-28.0)		
	Sedentary	22.00 (20.25-25.5)			17.00 (16.0-20.0)			26.00 (23.0-29.0)		
BMI category	Normal weight	17.00 (12.0-20.0)	H = 28.57	<0.001*	10.00 (4.00-16.0)	H = 23.55	<0.001*	18.00 (14.0-23.0)	H = 33.25	<0.001*
	Overweight	20.00 (17.0-24.0)			15.00 (10.0-19.0)			24.00 (19.0-27.0)		
	Obese	21.00 (20.0-24.0)			16.00 (13.0-20.0)			26.00 (22.0-29.0)		

Table 4 presents the adjusted odds ratios from the multivariate logistic regression models predicting the likelihood of experiencing symptoms of depression, anxiety, and stress (compared to normal levels), controlling for selected demographic, smoking, health, and lifestyle factors. These models indicate that the level of nicotine dependence is a highly significant independent predictor of symptoms across all three mental health subscales. The presence of chronic diseases also significantly predicted depression and anxiety symptoms. Monthly income (specifically the 3,000-6,000 SAR category) was significantly associated with lower odds of anxiety and stress symptoms compared to the lowest income group. Age and physical activity level (insufficiently active) were significant independent predictors only for stress symptoms in these models.

**Table 4:** Multivariate Logistic Regression Predicting Depression, Anxiety, and Stress Ref. is the reference category of the predictor. * Significant at p ≤ 0.05. Model Test refers to the Omnibus Tests of Model Coefficients, which assesses the statistical significance of the overall model. OR: adjusted odds ratio; CI: confidence interval.

Predictors	OR	95% CI for OR Lower	95% CI for OR Upper	P-value
Depression predictors				
Age	0.630	0.110	3.57	0.610
Years smoking	1.00	0.830	1.21	0.970
Dependence level	30.4	7.65	120.5	<0.001*
Chronic diseases (Ref: No)				
Chronic diseases (Yes)	13.81	1.24	154.10	0.033*
Model test χ²(4) = 72.11, p < .001				
Anxiety predictors				
Age	1.12	0.400	3.11	0.830
Living status (Ref: Alone)				
- With family	1.69	0.280	10.19	0.560
Monthly income (Ref: < 3,000 SAR)				
- 3,000–6,000 SAR	0.160	0.040	0.750	0.020*
- 6,001–10,000 SAR	0.220	0.040	1.13	0.070
- >10,000 SAR	0.600	0.060	6.29	0.670
Years smoking	1.10	0.980	1.24	0.110
Dependence level	22.08	6.910	70.57	< .001>
Chronic diseases (Ref: No)				
- Yes	4.99	1.590	15.65	0.006*
Vigorous activity days (Ref: 1 day)				
- 0 days	0.30	0.020	4.72	0.390
Physical activities classification (Ref: Active)				
- Insufficiently active	6.23	0.050	745.32	0.450
- Sedentary	9.92	0.180	541.25	0.260
BMI category (Ref: Normal weight)				
- Overweight	0.91	0.200	4.08	0.910
- Obese	0.38	0.050	2.91	0.350
Model test χ²(13) = 170.72, p < .001				
Stress predictors				
Age	1.13	1.096	1.176	0.004*
Living status (Ref: Alone)				
- With family	6.59	0.510	84.95	0.150
Monthly income (Ref: < 3,000 SAR)				
- 3,000–6,000 SAR	0.090	0.020	0.570	0.010*
- 6,001–10,000 SAR	0.230	0.030	1.64	0.140
- > 10,000 SAR	0.460	0.020	10.71	0.630
Years smoking	1.12	0.930	1.34	0.240
Dependence level	27.2	6.200	119.39	<0.001*
Vigorous activity days (Ref: 1 day)				
- 0 days	1.88	0.080	45.93	0.700
Physical activities classification (Ref: Active)				
- Insufficiently active	3.35	1.450	7.74	0.007*
- Sedentary	4.6	0.590	35.72	0.009*
BMI category (Ref: Normal weight)				
- Overweight	0.510	0.1	2.59	0.420
- Obese	0.210	0.01	4.24	0.310
Model test χ²(12) = 142.93, p < .001				

## Discussion

The purpose of this study was to analyze the association between mental health conditions and the level of nicotine dependence among adults who smoke cigarettes and were visiting PHCs in Taif City, Saudi Arabia. The results showed that participants had a high prevalence of depression (44.8% severe), anxiety (29.7% severe), and stress (38.1% severe); moreover, participants with greater dependence on nicotine had worse mental health conditions. These findings are consistent with the international literature documenting the existence of a two-way relationship between smoking and mental illnesses [3-5,21]. The data surrounding the sociodemographic characteristics of the study participants, along with the severity of these conditions, paint a different picture of Saudi Arabia, which is culturally and systemically healthcare-oriented, suggesting that some of these factors may moderate this phenomenon.

The stress and anxiety symptoms alongside depression reflect international findings. For example, Taylor et al. [4] revealed that smokers with depression were two to three times more likely to be dependent on nicotine, which is consistent with our results (OR = 30.36 for depression and high nicotine dependence). This is similar to the heightened anxiety (29.7% severe) that resonates with other works stating that smokers frequently utilize nicotine to self-medicate for anxiety syndromes and enter self-inflicted cycles of addiction and aggravated mental health [22,23]. At the same time, the mental health symptoms noted in this study are worse than what is reported in Western countries. For instance, Cerimele et al. [24] in the United States reported 28% of smokers in primary care as having moderate to severe depression. Our sample reported moderate, severe, or extremely severe depression at 84.2%. While these numbers appear stark, they may indicate a possible cultural concern, such as unemployment (9% in our sample) and financial strain (21% earning less than 3,000 SAR/month). These factors, which are known to deepen stress and aggravate associated smoking and mental health conditions in Saudi Arabia [6], represent a significant concern. Furthermore, there is underreporting of mental health service usage, which has previously been noted in Saudi Arabia [14], leading to unsupervised symptom aggravation among smokers.

The pronounced correlation between severe symptoms of mental illness and high nicotine dependence (FTND scores ≥7) reinforces the theory of the self-medication hypothesis [25], which suggests that smoking is often used to ease psychological suffering. Interestingly, those who smoked within five minutes of waking (indicating high dependence) had median depression scores of 23 (IQR: 21-25), while smoking after 60 minutes resulted in a score of 11 (IQR: 8.5-14.5). These results are consistent with earlier studies showing that rapid nicotine consumption increases dependence and exacerbates depressive disorders [26]. On the other hand, our finding that prior quit attempts were linked to lower depression scores contrasts with some literature. While Huddlestone et al. [26] reported that withdrawal may cause mood decline, leading to futile quit attempts, our results suggest that smokers who try to quit may have a healthier underlying mental state. Our data confirm findings from previous studies [26-28] showing a linkage between poor mental health and nicotine dependence. However, this association was not significant for smoking duration. The explanation behind the association between cigarette smoking and emotional disorders may be that smoking reduces one's capacity to tolerate stressful situations, causes maladaptive reactions, and dysregulates coping mechanisms to emotional states [29]. Further investigation is needed, as Saudi smokers may face different and dynamic barriers, such as restricted access to cessation medication like bupropion, that alter these relationships.

The consequences of mental health are significantly influenced by the socioeconomic status (SES) of an individual. Participants earning within the 3,000-6,000 SAR range had lower odds of suffering from anxiety (OR = 0.16) compared to their counterparts earning below 3,000 SAR. This is in line with global trends where low SES is associated with greater risks of mental health disorders and substance abuse, including smoking and alcohol use [30]. However, there was a weaker protective effect of higher income with regard to stress, indicating that non-financial, socio-culturally rooted expectations may contribute more significantly to stress in this group. Additionally, lifestyle factors were equally important. Sedentary participants reported higher median stress scores compared to their active peers, further supporting existing evidence that physical activity is an effective stress reliever [19,31]. The high prevalence of obesity (27.7%) reinforces evidence chronicled in Saudi Arabia’s ongoing dilemma of smoking coexisting with poor mental health and metabolic disease [32], underscoring the need for integrated interventions.

The coexistence of chronic diseases with mental health disorders poses an important public health problem. While hypertension was found in 29.6% of the participants, diabetes was also reported in 19%. The latter was associated with higher depression scores, which is in line with other studies conducted globally [33-35] and with another study done in Saudi Arabia by Hayes et al. [32]. The coexistence of these conditions is likely due to biological (inflammation) and psychosocial disease management stressors. Considering the prevalence of chronic diseases in Saudi Arabia [32,36], smoking cessation programs should address these risks.

The high percentage of male smokers (74.8%) reflects smoking patterns and practices within Saudi society, where male tobacco use is deeply rooted [7]. In contrast, the overwhelming mental health burden among women (25.2%) suggests that female smokers may experience additional societal stigma, which adds to psychological suffering. This is supported by Tobaiqy et al. [12], who reported that Saudi women smokers tend to minimize their mental health concerns to avoid negative perceptions. The limited use of bupropion further illustrates the gap in addressing this population. Despite international recommendations suggesting bupropion for depressed smokers in Saudi Arabia [3], the limited availability of this medication in Saudi PHCs is likely to worsen cessation outcomes. The underutilization of bupropion in Saudi Arabia’s smoking cessation programs might reflect significant systemic and cultural barriers. Clinically, many primary care providers lack training in pharmacotherapy for nicotine dependence, with most relying solely on behavioral counseling [12]. This knowledge gap may be compounded by bureaucratic hurdles, as bupropion often requires specialist approval and may not be routinely available in primary care formularies. Culturally, the medication may face stigma as a psychiatric treatment in a society where mental health care remains highly stigmatized, hindering the use of pharmacological interventions [14]. These barriers are particularly pronounced for women, who already represent a minority of smokers and face additional gender-related constraints in seeking treatment [12]. The lack of public awareness about bupropion’s dual mechanism (as both an antidepressant and smoking cessation aid) further limits its acceptance. These challenges persist despite strong evidence for bupropion’s efficacy, especially among depressed smokers who comprised nearly half of our study population [9,10]. Addressing this treatment gap will require coordinated efforts, including provider education, formulary policy changes, and public awareness campaigns to destigmatize medication-assisted cessation. Such measures could significantly improve outcomes for Saudi smokers, particularly those with comorbid mental health conditions who stand to benefit most from bupropion’s dual effects. Thus, this study highlights the need for relevant authorities in Saudi Arabia to revise policies by increasing the use of pharmacotherapy in addition to counseling.

This study’s strength stems from its consideration of the intersection between mental health and nicotine dependence among adult cigarette smokers in Taif, Saudi Arabia. It applies random sampling from multiple PHCs, which improves generalizability. The study uses validated instruments to assess mental health, nicotine dependence, and physical activity, and employs advanced statistical techniques to evaluate the associations. It also contributes to the existing regional literature by including the effects of socioeconomic status on smoking and mental health. The results urge the Saudi primary healthcare system to incorporate comprehensive mental health services alongside smoking cessation support, advancing healthcare in Saudi Arabia.

While PHC recruitment offers logistical advantages, the findings may not fully represent Saudi Arabia’s diverse smoker population. The observed associations between nicotine dependence and mental health should be interpreted with caution, particularly for underserved or non-help-seeking subgroups. In addition, the lack of causal explanations and self-report bias leading to underreporting are some of the study’s limitations. The sample was limited to attendees at Taif PHCs, excluding non-clinical populations, which, along with concerns about generalizability to the broader Saudi or international population, is problematic. Also, there were no clinical diagnoses provided; rather, mental health was screened using DASS-21 instead of direct interviews by clinicians. The limited female representation, as well as unaccounted confounding factors such as genetic predisposition, environmental stressors, or accessibility to mental healthcare, may further constrain the application of the findings. Therefore, clinical assessment validation and sampling from diverse populations should be integrated in future work to strengthen the research. These considerations may aid in constructing comprehensive mental health care and tobacco cessation programs based on the findings.

The exceptionally high odds ratios observed in this study (particularly the 30-fold increased odds of depression among participants with high nicotine dependence) warrant careful interpretation. While these estimates starkly highlight the strength of the association between severe nicotine dependence and poor mental health outcomes, several factors may contribute to their magnitude. First, the clinical nature of our PHC-based sample likely selected for smokers with greater health burdens, as individuals seeking care may represent more severe cases of both dependence and mental health symptoms. Second, the restricted variability in our reference groups (e.g., only 4.2% of participants had low nicotine dependence) may have amplified effect sizes due to sparse data in comparison categories. Third, residual confounding by unmeasured variables such as genetic risk factors, childhood trauma, or duration of untreated mental illness could further inflate these associations. While the dose-response gradient across dependence levels supports biological plausibility, the reported ORs should be viewed as extreme contrasts (high vs. low dependence) rather than incremental risks. These findings emphasize the profound mental health challenges faced by heavily dependent smokers in clinical settings, but population-based studies are needed to determine whether these effect sizes persist in broader community samples. Future research should incorporate advanced modeling techniques to account for potential collinearity and confounding, while clinical interventions should prioritize integrated treatment for this high-risk subgroup.

## Conclusions

This study reveals the relationship between the level of dependence on nicotine and mental health issues among adult smokers in Taif, Saudi Arabia, as well as the accompanying health problems such as depression, stress, and anxiety. Equally worrisome is the fact that those with stronger dependence on nicotine tend to have greater and more severe mental health problems than others. Of greater concern are those with chronic illness and low socioeconomic status, as they seem to suffer the most. Many are struggling with multiple chronic diseases, which take a toll on their mental well-being, while also dealing with financial constraints they can no longer afford to ignore. This study brings attention to the severe problems of mental illness and dependency on tobacco products in Saudi Arabia and the role of socioeconomics, lifestyle habits, culture, and the modern healthcare system. These findings highlight the need for integrated approaches encompassing psychological interventions, smoking or tobacco cessation services, and management of chronic health issues. Placing focus on women and increasing access to medications such as bupropion could help curb the cycle of smoking and mental health problems that so many in this population are caught in.

Future research should aim to establish causality and clarify the observed relationships through longitudinal studies that track smokers’ mental health and nicotine dependence trajectories over time. Intervention studies could test whether integrated treatment (e.g., bupropion combined with mental health support) improves outcomes compared to standard care. These designs would help disentangle whether severe dependence worsens mental health or vice versa, while addressing the high comorbidity identified in this study. Population-based sampling would further enhance generalizability beyond clinical settings.
